# Demographic Barriers for Genetic Testing in High-Risk Breast Cancer Patients in the Northern Michigan Area

**DOI:** 10.7759/cureus.22966

**Published:** 2022-03-08

**Authors:** Danielle Hebert, Felipe Pacheco, Lisa WintonLi, Asma Taj

**Affiliations:** 1 General Surgery, Central Michigan University College of Medicine, Saginaw, USA; 2 Breast Surgery, Central Michigan University College of Medicine, Saginaw, USA; 3 Medical Oncology, Ascension Health, Saginaw, USA

**Keywords:** barriers to genetic testing, genetic counseling, genetic testing, breast cancer, breast

## Abstract

Introduction: The National Comprehensive Cancer Network (NCCN) has outlined guidelines for criteria regarding genetic testing for high-penetrance breast and/or ovarian cancer susceptibility genes. Due to the lack of availability of genetic counseling services in Northern Michigan prior to COVID-19, the utilization of genetic testing falls well below recommended guidelines.

Methods: Patients diagnosed with breast cancer in 2019 were randomly selected from Ascension Michigan's Northern Ministries Tumor Registry. A retrospective chart review was conducted. For patients who met NCCN criteria, their medical records were used to determine if genetic testing was recommended and if genetic testing was completed. Univariate (Crosstabs and t-tests) and multivariate tests with logistic regression were used to identify significant associations between the variables of interest.

Results: One hundred and two (102) patient charts were reviewed in this group; 55 (52.4%) were eligible by the NCCN guidelines for genetic testing. From this eligible subset of patients, only 29 were offered genetic testing, and only 21 were tested. The mean age of the patients offered genetic counseling was 56.2 years compared and 67.6 years in the group not offered counseling (p < 0.001). The patient's insurance type was an independent factor for obtaining genetic testing, specifically, the subgroup who had Medicare (OR = 0.73, CI = 0.01-0.54; p = 0.02). Patients insured through Medicare were less likely to obtain genetic testing after referral to a genetic counselor (p = 0.01).

Conclusion: Genetic counseling for high-risk breast cancer patients is below average in Northern Michigan, likely related to lack of physician referral, poor availability of counseling services, low socioeconomic status as well as a lower level of concern in older ages.

## Introduction

Breast cancer is the leading cause of cancer diagnoses among women worldwide (excluding skin cancer) [1]. Approximately 5% to 10% of breast cancers have a hereditary component [2]. Due to the high prevalence of breast cancer worldwide and the significant hereditary component, the National Comprehensive Cancer Network (NCCN) has outlined specific criteria for genetic counseling for patients with or at considerable risk for breast cancer. The NCCN clinical practice guidelines in oncology for Genetic/Familial High-Risk Assessment: Breast, Ovarian, and Pancreatic make recommendations for patients based on several factors, including age at diagnosis, sex, personal and family history of cancer (NCCN version 1.2020) [3]. The American Society of Breast Surgeons supported the need for genetic counseling in hereditary breast cancer in patients with and without a personal history of breast cancer who meet NCCN criteria in their February 2019 consensus guidelines [4]. Numerous professional societies support genetic counseling for patients who meet criteria as results may alter the frequency of surveillance and treatment protocol and have implications for future generations.

Genetic counseling has become more robust since it first became available. The first major utilization of genetic counseling was for classifying a patient’s BRCA status [5,6]. Genetic counseling begins with evaluating a patient’s risk for certain types of cancer, based upon their personal medical history and family cancer history [7,8]. From here, a decision can be made to pursue genetic testing or defer testing based on the level of risk and the clinical relevance the results may have on treatment and screening protocols. Today, genetic testing services provide multi-gene panels that identify several genes associated with breast cancer in addition to genetic variants of unknown significance [5,9]. The goal of counseling after genetic testing is shifted to providing patients with recommendations and education on the clinical significance of their results.

The value that genetic counseling provides is undeniable, and the need for this counseling has heightened as genetic testing has advanced. One way in which genetic counseling services have catered to this need is through the implementation of virtual genetic counseling. Virtual genetic counseling is particularly useful in rural communities, as in-person services are most often located in academic and urban settings [8]. One study found that BRCA1/2 counseling over the phone was as effective as in-person counseling in regard to providing education and informed decision-making [10]. Despite the availability of many resources, including virtual services, genetic counseling services have consistently been underutilized among many patient populations [2,11,12]. This may be due to a lack of awareness of genetic counseling or its implications, having limited knowledge of their family history or personal risk, or having concerns regarding the financial burden of counseling and subsequent testing [11,13,14].

Several factors contribute to the lack of referral to a genetic counselor in patients who meet NCCN criteria. The purpose of this study is to identify barriers to genetic counseling for patients in the Northern Michigan region to begin recognizing ways to overcome genetic counseling discrepancies.

## Materials and methods

Study design

After approval from the Ascension St. Mary’s Institutional Review Board (IRB) was obtained, a retrospective analytical study based on the hospital records of patients diagnosed with breast cancer in the Northern Michigan area was conducted.

Patients diagnosed with breast cancer in 2019 were selected from the Ascension Michigan’s Northern Ministries Tumor Registry. Using the NCCN guidelines (NCCN version 1.2020), patients eligible for genetic testing were identified based on their individual risk factors. Of those who met NCCN criteria, two subsequent groups were made: patients who were recommended to have a genetic counseling referral and patients who were not. The proportions of patients who underwent testing and those who did not obtain testing following a recommendation by a genetic counselor were also evaluated. Several demographic variables were also obtained through chart review to assess potential contributing factors to a patient’s decision to undergo genetic testing. 

Inclusion and Exclusion Criteria

Patients who were diagnosed with breast cancer within 2019 were included. A subgroup of 55 patients was obtained after including only the patients eligible for genetic counseling and testing per the NCCN guidelines. The excluded patients were those with incomplete documentation in their medical records that would inhibit accurate evaluation of the fulfillment of NCCN criteria or those with equivocal data regarding genetic counseling.

Statistical analysis

The statistical analyses were run using the IBM SPSS Statistics for Windows software (version 26.0; IBM Corp., Armonk, NY). Frequency tables were used for univariate analysis of the demographic data. To adjust for covariates, a simple logistic regression model was used. All the statistical tests were two-sided, and p < 0.05 indicated a statistically significant difference.

## Results

Population demographics

At the conclusion of the retrospective review, 105 patients from the Northern Michigan area Ascension Tumor Registry were identified. Of those, 102 were selected as they had fully documented medical records. In this group, 55 (52.4%) were eligible for genetic counseling referral by the 2020 NCCN guidelines (Table 1). However, only 29 of the 55 eligible patients were offered a referral to a genetic counselor. The racial data for the patient population used was as follows: 89% identified as white, 6.7% as black, and 3.8% identified as other. The geographic distribution of the included population was as follows: 40% resided in a rural area (defined as a county population < 50,000) while 60% lived in an urban area (defined as a county population > 50,000). Regarding patient insurance, 47.3% had Medicare, 36.4% had private insurance, 3.6% had Medicaid, and the rest had undisclosed insurance in the chart review. The age at breast cancer diagnosis ranged from 36 to 87 years, with a mean of 64 years.

**Table 1:** Demographic Date for NCCN Eligible Patients *Urban defined by > 50,000 county population.

Variable		n	%
Sex	Female	54	98.1
	Male	1	1.9
Race	White	49	89.1
	Black	4	7.3
	Other	2	3.6
Insurance	None	0	0
	Medicaid	2	3.6
	Medicare	26	47.3
	Private	20	36.4
	Insured, unspecified	7	12.7
Residence	Urban*	22	40.0
	Rural	33	60.0

Genetic testing offered 

Of the 55 (52.4%) patients eligible by NCCN criteria for genetic counseling, only 29 were offered a referral to a genetic counselor. However, only 21 (72.4%) of the patients offered referral to a genetic counselor went on to obtain genetic testing. No significant difference was found based on race (p = 0.9) among those who were offered genetic counseling and those who were not. The mean age of patients offered genetic counseling was 56.2 years compared to 67.6 years in the group that was not offered a referral (p < 0.001). When comparing if genetic counseling was offered based on a patient’s insurance and their permanent residence (rural vs. urban), there was no significant difference based on insurance type and geographic location (p = 0.22, p = 0.37, respectively). On the multivariate logistic regression, there was no significant variable associated with decreased or increased offering of genetic counseling in breast cancer patients. 

Genetic testing obtained

The subset of patients in whom genetic counseling was offered was further divided into patients who went on to obtain genetic testing and those who did not. Again, age was a significant factor in the univariate analysis for obtaining genetic testing (p < 0.001), with younger patients more likely to utilize genetic testing resources. When comparing race or residence (rural vs. urban), once again, no significant difference between the two groups was identified (p = 0.48, p = 0.09, respectively). The only significant variable on multivariable analysis that was associated with decreased genetic testing performed was the insurance type (OR = 0.73, CI = 0.01-0.54; p = 0.02). Specifically, being insured through Medicare was seen more frequently among those who did not obtain testing after being offered (p = 0.01). 

## Discussion

Genetic counseling and testing have revolutionized the treatment and management of breast cancer in the late 1990s and have continued to advance in the following years. As technology has advanced and the number of genetic variations that testing is capable of identifying has improved, a greater proportion of the patient population has been able to utilize these resources [6,15]. NCCN criteria have continually evolved, and with it, eligibility criteria [15]. However, despite the decreased cost of genetic counseling and testing, broadened eligibility criteria, and improved availability of services, many patients still fail to obtain genetic counseling and, therefore, testing. 

Several studies have attempted to identify barriers to obtaining genetic counseling. One study in Dallas, Texas, reported 69% of patients who were eligible by NCCN criteria for breast cancer genetic testing obtained it. The most significant barrier to genetic testing in that study was the lack of a timely referral from a physician to a genetic counselor [2]. Several factors may influence physician referral, including physician awareness of the services available to their patients and/or inaccessibility of such services for a given geographic location [2,13,14].

In another study, only 43.5% of patients with a clinical indication for genetic testing received a referral to a genetic counselor [12]. Across the United States, there is a shortage of cancer-trained genetic counselors, which undoubtedly contributes to this underutilization of genetic testing [5]. A physician's awareness of available resources for their patients is a potential contributing factor to this discrepancy of genetic counseling referral. Of breast cancer patients who meet NCCN criteria, 47.3% (n=26) were not offered counseling. Although a specific explanation for each patient not being offered counseling cannot be provided, there are still a few likely scenarios that can be deduced. Most notably, the lack of available resources for genetic counseling in the Northern Michigan area and a lack of knowledge of virtual counseling services. 

The patient's age had a significant influence on being offered genetic counseling in this study. Younger patients more commonly had discussions with their physicians regarding genetic counseling than older patients with newly diagnosed breast cancer. It can be inferred that the younger the patient, the more likely the patient is to be offered counseling because, classically, breast cancer associated with genetic mutations occurs at a younger age [16]. Older patients are often mistakenly overlooked for genetic counseling referrals, and this may have detrimental consequences for both the patient and future generations. 

Although not demonstrated in this study, race has been identified as a barrier to genetic counseling in previous studies [17]. Further studies in this geographic region with a larger sample size are encouraged to characterize the role of race more clearly as a barrier to genetic counseling services. 

Patient demographics and variables that predicted a patient's likelihood of obtaining testing after being offered counseling were also identified. The urban versus rural delineations did not influence whether a patient obtained testing. This clearly demonstrates the underutilization of breast cancer genetic counseling services for the entire Northern Michigan area. Patients insured through Medicaid were less likely to get genetic testing. Insurance coverage should not be a limitation for patients who meet NCCN criteria because under the Affordable Care Act, genetic counseling, and specifically BRCA testing, are recommended preventative services for women found to be at increased risk for hereditary breast and ovarian cancer [18]. Therefore, it is not the insurance carrier itself that contributes to the follow-through with counseling, rather the socioeconomic and educational backgrounds of patients. Patient education is another important determinant of genetic counseling services. The motivation to obtain testing originates from an understanding of the implications of finding genetic mutations. These implications include alterations in treatment protocol, frequency of future monitoring, risk of secondary malignancies, and risk of cancer development in family members [16]. It is the responsibility of the physician who orders the testing and the genetic counselor to educate the patient, giving special attention to the importance of the results of genetic testing in addition to the risks, benefits, and limitations of testing. Lower socioeconomic status contributes to an inability to afford the travel to the available genetic counseling location and/or lack of resources to follow through with virtual counseling. Virtual genetic counseling services have been available for years; however, with the COVID-19 pandemic, the benefits of virtual counseling have become very apparent [19]. The importance of recognizing patients who cannot carry this financial burden is crucial because they are at risk of being lost to follow-up. Resources remain available to those who require extra financial assistance. 

## Conclusions

Genetic counseling should be offered to all patients who fulfill NCCN criteria regardless of their socioeconomic status, race, and geographic location. Virtual genetic counseling services paired with at-home genetic testing kits have increased accessibility to testing and the means to educate patients on the clinical relevance of the results. Continuing to advocate for widespread genetic counseling services for all newly diagnosed breast cancer patients who meet NCCN criteria is crucial.

With the incorporation of virtual genetic counseling, it may be anticipated that the discrepancy in genetic counseling utilization will lessen in the coming years. Future comparative analysis may look at the proportion of patients who meet NCCN criteria for referral to a genetic counselor pre- and post-COVID-19. The major limitation of this study is that it employed a retrospective chart review which relies on the assumption that the patient documentation was complete at the time of review. The sample size is also a limitation and should be continued with a larger patient population to fully address discrepancies in genetic counseling referral.
